# Supplementary material for: Modelling Skylarks (Alauda arvensis) to Predict Impacts of Changes in Land Management and Policy: Development and Testing of an Agent-Based Model
Source: PLoS One. 2013 Jun 6;8(6):e65803. doi: 10.1371/journal.pone.0065803 (PMC3675089; doi:10.1371/journal.pone.0065803)
Supplement: Supporting Information S4 — The skylark ODdox as a zipped archive. (ZIP) [file pone.0065803.s004.zip › Skylark_ODdox/globals_0x67.html]

ALMaSS Skylark ODdox: File Members


ALMaSS Skylark ODdox
 2.0


- Main Page
- Related Pages
- Classes
- Files

- File List
- File Members

- All
- Functions
- Variables
- Typedefs
- Enumerations
- Enumerator
- Macros

- \_
- a
- b
- c
- d
- e
- f
- g
- h
- i
- j
- k
- l
- m
- n
- o
- p
- r
- s
- t
- u
- v
- w
- x

Here is a list of all file members with links to the files they belong to:

### - g -

- g\_ALandscape
  : ALMaSS\_CmdLine.cpp
- g\_AManager
  : ALMaSS\_CmdLine.cpp
- g\_biomass\_scale
  : elements.cpp
- g\_bug\_percent\_a
  : elements.cpp
- g\_bug\_percent\_b
  : elements.cpp
- g\_bug\_percent\_c
  : elements.cpp
- g\_bug\_percent\_d
  : elements.cpp
- g\_cfg
  : configurator.cpp
  , configurator.h
- g\_chance\_gen01000
  : ALMaSS\_CmdLine.cpp
- g\_chance\_gen010000
  : ALMaSS\_CmdLine.cpp
- g\_crops
  : plants.cpp
  , plants.h
- g\_date
  : calendar.cpp
  , calendar.h
- g\_el\_herbicide\_delaytime\_days
  : elements.cpp
  , elements.h
- g\_el\_strigling\_delaytime\_days
  : elements.cpp
  , elements.h
- g\_el\_tramline\_decaytime\_days
  : elements.cpp
  , elements.h
- g\_farm\_enable\_crop\_rotation
  : map\_cfg.cpp
  , map\_cfg.h
- g\_farm\_fixed\_crop\_enable
  : map\_cfg.cpp
  , map\_cfg.h
- g\_farm\_fixed\_crop\_type
  : map\_cfg.cpp
  , map\_cfg.h
- g\_farm\_fixed\_rotation\_enable
  : map\_cfg.cpp
  , map\_cfg.h
- g\_farm\_fixed\_rotation\_farms\_async
  : map\_cfg.cpp
  , map\_cfg.h
- g\_farm\_fixed\_rotation\_farmtype
  : map\_cfg.cpp
  , map\_cfg.h
- g\_farm\_test\_crop\_daystorun
  : map\_cfg.cpp
  , map\_cfg.h
- g\_farm\_test\_crop\_farmtype
  : map\_cfg.cpp
  , map\_cfg.h
- g\_farm\_test\_crop\_filename
  : map\_cfg.cpp
  , map\_cfg.h
- g\_farm\_test\_crop\_reportdaily
  : map\_cfg.cpp
  , map\_cfg.h
- g\_farm\_test\_crop\_sample\_x
  : map\_cfg.cpp
  , map\_cfg.h
- g\_farm\_test\_crop\_sample\_y
  : map\_cfg.cpp
  , map\_cfg.h
- g\_farm\_test\_crop\_type
  : map\_cfg.cpp
  , map\_cfg.h
- g\_files
  : ALMaSS\_CmdLine.cpp
- g\_generator()
  : ALMaSS\_CmdLine.cpp
- g\_land
  : skylarks\_all.cpp
- g\_landscape\_p
  : elements.h
  , farm.cpp
  , farmfuncs.cpp
  , Landscape.cpp
- g\_letype
  : elements.cpp
  , elements.h
- g\_map
  : landscape.h
- g\_map\_le\_border\_chance
  : map\_cfg.cpp
  , map\_cfg.h
- g\_map\_le\_borders
  : map\_cfg.h
  , map\_cfg.cpp
- g\_map\_le\_unsprayedmargins
  : map\_cfg.cpp
  , map\_cfg.h
- g\_map\_le\_unsprayedmargins\_chance
  : map\_cfg.cpp
  , map\_cfg.h
- g\_map\_maxpolyref
  : map\_cfg.cpp
  , map\_cfg.h
  , skylarks\_all.cpp
- g\_map\_orchards\_borders
  : map\_cfg.h
  , map\_cfg.cpp
- g\_map\_orchardsborderwidth
  : map\_cfg.cpp
  , map\_cfg.h
- g\_msg
  : maperrormsg.h
  , maperrormsg.cpp
- g\_NoOfPredProbes
  : ALMaSS\_CmdLine.cpp
- g\_NoProbes
  : ALMaSS\_CmdLine.cpp
- g\_pest
  : pesticide.h
  , pesticide.cpp
  , landscape.h
- g\_PredatorManager
  : ALMaSS\_CmdLine.cpp
- g\_Predfiles
  : ALMaSS\_CmdLine.cpp
- g\_PredResultsDir
  : ALMaSS\_CmdLine.cpp
- g\_rand\_uni()
  : ALMaSS\_CmdLine.cpp
  , Rodenticide.cpp
  , skylarks\_all.cpp
- g\_randmaxp
  : ALMaSS\_CmdLine.cpp
- g\_ResultsDir
  : ALMaSS\_CmdLine.cpp
- g\_rotation
  : croprotation.cpp
  , croprotation.h
- g\_Species
  : ALMaSS\_CmdLine.cpp
- g\_SpeedyDivides
  : Landscape.cpp
- g\_Steps
  : ALMaSS\_CmdLine.cpp
- g\_stopdate
  : skylarks\_all.cpp
- g\_str
  : PopulationManager.cpp
- g\_time
  : ALMaSS\_CmdLine.cpp
- g\_torun
  : ALMaSS\_CmdLine.cpp
- g\_uni\_dist()
  : ALMaSS\_CmdLine.cpp
- g\_weather
  : weather.h
  , weather.cpp
- g\_weed\_percent
  : elements.cpp
- g\_Year
  : ALMaSS\_CmdLine.cpp
- gen\_type\_int
  : BoostRandomGenerators.h
- GetProbeInput\_ini()
  : ALMaSS\_CmdLine.cpp
- glyphosate
  : treatment.h
- Growth\_Phases
  : plants.h
- growth\_regulator
  : treatment.h


- Generated on Thu Jan 10 2013 13:15:39 for ALMaSS Skylark ODdox by
   1.8.1.1
